# Effect of dietary supplementation of Sargassum meal on laying performance and egg quality of Leghorn layers

**DOI:** 10.5713/ajas.20.0256

**Published:** 2020-08-21

**Authors:** Geng-Jen Fan, Bor-Ling Shih, Hui-Chiu Lin, Tzu Tai Lee, Churng-Faung Lee, Yih-Fwu Lin

**Affiliations:** 1 Nutrition Division, Livestock Research Institute, Council of Agriculture (COA), Hsinhua, Tainan, 712009, Taiwan; 2 Department of Animal Science, National Chung Hsing University, Taichung 402204, Taiwan; 3 Penghu Marine Biology Research Center, Fisheries Research Institute, Council of Agriculture, Executive Yuan, Makung, Penghu 880010, Taiwan

**Keywords:** Egg Quality, Leghorn Layers, Laying Performance, Sargassum Meal

## Abstract

**Objective:**

Seaweeds could be an alternative and functional feed resource. The purpose of this experiment is to investigate the effect of dietary supplementation of Sargassum meal on laying performance and egg quality of layers.

**Methods:**

Two hundred 36-wk-old layers were divided into five treatment groups. Each treatment had four replicates with 10 hens per experimental unit. The corn-soybean meal basal diet was formulated as control group. Sargassum meals were included 0%, 1%, 2%, 3%, or 5% to diets for five treatment groups, respectively. Treatment groups were isocaloric-isonitrogenous diets. Laying performance and egg quality were measured for eight weeks.

**Results:**

Sargassum meal supplementation did not affect daily feed intake. Supplementation 1% to 3% of Sargassum meal in diets increased daily laying rate and egg mass compared with those from control group (p<0.05). Egg qualities among five groups were all similar. Supplementation of 3% Sargassum meal increased the lightness of egg yolk (p<0.05). Eggs produced from layers fed 1% and 2% Sargassum meal had a higher consumer’s acceptability than the control group (p<0.05). In blood characteristics, contents of glucose, nitrogen, triiodothyronine (T3) and thyroxine (T4) increased as the increase of supplementation ratio of Sargassum meal (p<0.05). In serum antibody titers, supplementation of 2% Sargassum meal stimulated a higher immunoglobulin M (IgM) level than that from control group (p<0.05). However, IgM content of layers fed diets with Sargassum meal ≥3% were decreased (p<0.05). There was no difference in IgA and IgG titers among groups.

**Conclusion:**

Supplementation of 1% to 3% Sargassum meal has shown to increase egg laying rate and egg mass of Leghorn layers. However, high supplementation (5%) would negatively affect laying performance. In consideration of laying performance, egg quality, consumer responses, and blood antibody, supplementation of Sargassum meal was suggested 2% in the diet for layers.

## INTRODUCTION

Seaweeds are photosynthetic algae and abundant in all sea areas of the earth. Seaweeds are also rich in vitamins, minerals and bio-activators which can promote the growth of aquatic fish [1]. Traditionally seaweeds can be used as food, folk remedies, dyes, and fertilizers. There are three main classes, or phyla, of seaweed: Phaeophyceae (brown algae), Rhodophyta (red algae), and Chlorophyta (green algae). Thousands of species comprise each phylum [2].

Taiwan is surrounded by the sea and the marine resources are quite rich. Penghu Islands have the most abundant seaweed production resources in Taiwan area. In recent years, diversified development of feed resources has become an international trend, especially macroalgae or seaweed. Due to high yield, it becomes an important source of protein for aquatic and terrestrial livestock.

Sargassum (*Sargassum hemiphyllum* var. *Chinense* J. Agardh) is a genus of brown algae notable for its economic importance due to its use in aquaculture. Sargassum is rich in sulfated polysaccharides - fucans which have antioxidant, antitumor, immunostimulatory, and anti-inflammatory functions [3]. Research of Lin et al [4] showed that Sargassum meal after treatment can have good antioxidant effects with total phenolics 10.45±0.77 mg/mL, 1,1-diphenyl-2-picrylhydrazyl 85.49%±2.10%, chelating ability 16.34%±4.91% and superoxide anion scavenging activity 40.77%±4.30%.

How to use the low-cost and functional seaweed as one feed ingredient has become an important research topic [5]. The Sargassum used in this experiment is the dominant, year-round growing and largest (1 to 2 m long) seaweed in Penghu Islands. The purpose of this experiment is to investigate the effects of dietary supplementation of Sargassum meal on laying performance and egg quality of Leghorn layers.

## MATERIALS AND METHODS

### Animal care

The trial was conducted in the experimental chicken barn in the Livestock Ressearch Institute (LRI) at Hsinhua, Tainan, Taiwan. All experimental procedures were approved by the Institutional Animal Care and Use committee (IACUC No. 104-27) of LRI.

### Preparation of Sargassum meal

Sargassum was mainly collected from the seashore of Magong city, Penghu. After collection, Sargassum was made by a process of classification, washing with tap water, dehydration with dehydrator and air drying with 70°C for 24 hours. After drying, Sargassum was ground into powder. Twenty liters of acetate buffer solution (0.1 N, pH 4.5) were added with 0.2% Viscozyme (including Arabanase, celluiase, b-glucanase, hemi-cellulase, and xyianase) and mixed with 10 kg Sargassum meal. After 12 hours of enzyme hydrolysis, it was air dried with 70°C and then ground to powder again, becoming the Sargassum meal. Nutrient contents of Sargassum meal were analyzed (as fed basis): moisture 6.48%±0.01%, crude ash 25.71%±0.33%, crude protein 12.29%±0.01%, crude fat 0.8% ±0.04%, and carbohydrate 54.72%±0.02%. Carbohydrate (%) was calculated as: 100 – moisture (%) – protein (%) – fat (%) – ash (%) – crude fibre. The carbohydrate contents of Sargassum meal were analyzed: neutral detergent fiber (NDF) 41.55%±0.38%, acid detergent fiber (ADF) 39.51%±0.16%, acid detergent lignin (ADL) 7.19%±0.40%, hemicellulose (NDF – ADF) 2.35%±0.22%, and cellulose (ADF – ADL) 32.32%±0.56%.

### Experimental animals and treatment

A total of two hundred Leghorn layers (Hy-line W-36) at 36 weeks of age were divided into five treatment groups. Each bird was housed in an individual cage measuring 23 cm×42 cm×40 cm (width×length×height) in an open-sided house. The experiment involving the laying period was conducted during Taiwan’s hot season (July to August). Each treatment group, containing 40 birds, was equally separated into four experimental units (replicates). A corn-soybean meal basal diet was used as the control group. The Sargassum meals coming from the Penghu Marine Biology Research Center were included 0%, 1%, 2%, 3%, or 5% to five treatment diets, respectively. The hens did not receive any vaccines or drugs during the entire experimental period. Feed and water were provided *ad libitum* with a light regimen of 17 h of continuous light per day in open-sided housing. The experiment was conducted for 8 weeks. Diets for each group were prepared isocaloric-isonitrogenous and formulated to contained 17% crude protein and 2,850 kcal/kg metabolizable energy. The other nutrients were formulated according to the nutrient requirement of laying hens of NRC [6] (Table 1).

### Measurements

i) Body weight was measured at the beginning and end of the experiment to determine the weight change of hens.

ii) Egg weight was measured two days a week and feed consumption was recorded every week. The number of eggs produced, and abnormal egg count was recorded every day. The hen day laying rate, egg mass and feed conversion ratio were calculated as follows:

$$\begin{array}{l} \text{Laying rate }(\%)=(\text{total number of eggs produced}/\text{number of hens}/\text{days of egg production})\times 100\\ \text{Egg mass per hen per day }(\text{g}/\text{d})=(\text{number of eggs produced}\times \text{average egg weight)}/\text{days of egg production}\\ \text{Feed conversion ratio}=\text{feed intake per hen per day}/\text{egg mass per hen per day} \end{array}$$

iii) Egg quality: Twenty eggs were collected per group every two weeks for measurement of egg quality and Haugh unit.

a. Eggshell quality determination included eggshell strength, eggshell thickness, and the percentage of eggshell weight/egg weight. The Hung-Ta (HT-8116) table-type compression strength tester was used to determine the strength of eggshell. A piece of eggshell at the blunt end, tip and equatorial part of the egg were taken and measured using micro measuring instrument to a decimal point of 3 digits. The average of the three measurements was the thickness of eggshell [7].
b. The percentage of eggshell weight was calculated according to the eggshell weight and egg weight.
c. Haugh unit measurement: After breaking the shell, the egg was placed on a horizontal egg quality gauge (FHK, Tokyo, Japan) and the height of the thick albumin was measured. Haugh unit was calculated by Haugh formula [8] according to the albumin height and egg weight.

$$\begin{array}{l} \text{Haugh unit}=100\times \text{log }(\text{H}-1.7\;{\left(\text{W}\right)}^{0.37}+7.6)\\ \text{H}=\text{albumin height }(\text{mm});\text{W}=\text{egg wight }(\text{g}) \end{array}$$

iv) Yolk color measurement: Yolk color was measured using colorimeter (Minolta Chroma Meter, Tokyo, Japan) [9]. Every 4 weeks, 20 eggs from each group were collected. Egg yolk was used to determine the brightness and color with 2 replicates.

v) Panel test: Evaluation items were defined before panel test. Eggs were cooked in 100°C water for 10 to 15 minutes. Eggs with well-done yolk were cut into 1/4 slices and stored in a 60°C oven till the evaluation. Twenty-four evaluators among 23 to 65 years of age scored each egg sample four items including flavor, color, tenderness, and total acceptancy. Score 7 was the best, score 1 the worst [10].

vi) Six hens from each group were randomly selected for blood test at the end of the experiment. Five mL of blood samples were taken from the wing vein. Serum was separated by the Centrifugal Separator (1,7000g, 15 min) and stored in a −20°C freezer for analysis. Glucose, total protein, urea nitrogen, total cholesterol and triglycerides content were analyzed using automatic analyzer (Hitachi 7176A, Tokyo, Japan) [11]. Triiodothyronine (T3) and thyroxine (T4) were analyzed using CMIA (Abbott Architect kit, Chemiluminescent Microparticle Immunoassay Assay, Abbott Park, IL, USA). Immunoglobulin (IgA, IgG, and IgM) were determined by using chicken ELISA quantitation set (Bethyl Laboratories Inc., Montgomery, TX, USA).

vii) Approximate nutrient contents of experimental diets: two diet samples were sent and analyzed at the Feed Analysis Center, Animal Nutrition Division, LRI using AOAC [12] methods.

### Statistics analysis

Trait data from the experiment was analyzed using the SAS 9.4 (SAS Institute Inc., Cary, NC, USA) with completely randomized design. For the statistical analysis of feed intake, feed conversion rate, egg mass and laying rate, average performance of every replicate with 10 birds was treated as an experimental unit. Before analysis, all percentage data were transformed to arcsine of the square root to normalize data distribution. Because no statistical differences were observed between the transformed and not transformed, the statistics presented in this paper were from the untransformed data. General linear model procedure was used for variant analysis. The model used was Y_ij_ = μ+T_i_+E_ij_. Where μ is the average value, T_i_ is the treatment value, and E_ij_ is the error value. If there was significant difference, the least squares mean was adopted for difference analysis among groups. The significant difference level was set p<0.05. Scoring data from panel test were analyzed the difference by Chi-square test [13].

## RESULTS AND DISCUSSION

### Egg production performance

Effects of dietary supplementation of Sargassum meal on feed intake and laying performance of layers are shown in Table 2. During 8 weeks of experimental period, isocaloric-isonitrogenous diets including Sargassum meal did not affect daily feed intake of hens. Layers fed on diet supplemented with 2% Sargassum meal had a higher hen-day egg production than that of the control and 5% groups (p<0.05). Groups given dietary supplementation of 1% and 3% Sargassum meal had produced a higher egg mass than those from the control and 5% groups (p<0.05). Feed conversion ratio (feed intake/egg mass) declined in the 5% supplementation group. All the Sargassum meal supplemented groups had a numerically lower body weight gain than the control group. There has been prior discussion regarding the proper level of seaweed added in poultry diet. In poultry rations, replacing the conventional proteins by algae up to a level of 5% to 10% could be used safely. However, prolonged feeding of algae at higher level might cause adverse effects [14]. Research indicated that the use of macroalgae such as Ulva and Sargassum should be restricted in poultry diets because of the low protein and fat content, indigestible polysaccharide, and low gross energy [15]. From this study, supplementation of moderate amount (1% to 3%) of Sargassum meal in diet could promote the laying performance, but an excess amount (5%) caused a negative effect.

### Egg weight and egg quality

Effects of dietary supplementation of Sargassum meal on egg quality and color of egg yolk of layers are shown in Table 3. Supplementation of Sargassum meal did not affect the egg weight, eggshell breaking strength, eggshell thickness, albumin height, shell weight percentage, and Haugh unit. However, supplementation of 3% Sargassum meal increased the lightness of egg yolk compared with that of the control and 1% groups (p<0.05). There was also the trend that supplementation of Sargassum meal could increase the a value (redness) and b value (yellowness) of yolk. It showed that supplementation of Sargassum meal (2% to 3%) could improve the lightness of egg yolk. In 3% group, daily feed intake was 98.13 g, that is, 2.94 g sargassum meal. In 5% group, daily feed intake was 91.3 g, that is, 4.57 g sargassum meal. In other words, although the 5% group had higher sargassum meal intake, lower total feed intake caused lower corn intake. Hence, decrease of lightness, redness, and yellowness in 5% group was probably due to the drop of feed intake, ie decrease of corn intake may overwhelm the sargassum meal effect on lightness.

Egg contains β-carotene, zeaxanthin, and β-apo-8-carote noic acid ethyl ester. Eggs and poultry meat are likely to be the primary contributors to the human intake of β-apo-8-carotenoid acid ethyl ester [16]. Absorption and accumulation of natural pigments significantly enhance the nutrition value and color of egg yolks and chicken [17]. Color of egg yolk can be deepened by the accumulation of carotenoids, including carotene and xanthophyll (cryptoxanthin, lutein, and zeaxanthin). Previous reports indicated that forage, alfalfa, chlorella, corn, marigold flower, red pepper, cabbage, and tangerine peel can be used to deepen yolk color [18–21]. In previous study [22], dietary corn totally replaced by rice could restore the yellow color of the egg yolk when natural lutein was added into diets. The result was similar to the research indicating that dietary grains rich in lutein, seaweed or crab shell could increase the yellow color of yolk [16,23]. Carrillo et al [24] reported that dietary supplementation with Sargassum may increase yolk color affects favorably the yolk color. Sargassum sp. has bioactive compounds that have a potential antioxidant activity such as phenolic compounds as well as chlorophyll and carotenoids [25]. Islam et al [26] indicated that correlation between L* and certain carotenoids was negative because increase in pigment was related to darkness; thereby decrease L*. Hence, the colour value a* (colour direction in red or green) was highly correlated with total carotenoid content. In this study, the supplementation of Sargassum meal tended to increase the yolk color, especially effective for the 3% group.

### Panel test of well-done eggs

Effects of Sargassum meal supplementation in diets on the panel test of eggs are shown in Table 4. Flavor score and tenderness scores were similar among groups. Supplementation of 2% Sargassum meal had higher color score than the other groups (p<0.05). Eggs produced from control group, 1% and 2% supplementation groups had the higher acceptance percentage (p<0.05). Supplementation of 5% Sargassum meal had negative effect on total acceptability thereafter affected the purchase intention (p<0.05). Eggs produced from 2% Sargassum meal group had the greatest acceptance. The purchase intention was over 60%. Results from this study indicating that faded yolk color obviously affects the consumer’s purchase willingness, agreed with Su [16] who concluded that deeper yolk color can increase purchase willingness. Chickens cannot synthesize carotenoids on their own, so they must be replenished with pigment ingredients from feed sources [27,28]. Research indicated that restoring the yolk color with lutein additives can improve the egg color, consumer’s acceptance and purchase willingness [22,28].

### Blood characteristics

Effects of Sargassum meal supplementation on blood characteristics of layers are shown in Table 5. The contents of blood glucose, blood urea nitrogen, T3 and T4 increased as the increase of dietary Sargassum meal supplementation. The group supplemented with 5% Sargassum meal had higher blood glucose, blood urea nitrogen, T3, and T4 than the control group (p<0.05). The results indicated that seaweeds like Sargassum meal might promote body metabolism then affect blood characteristics of hens. Previous studies regarding algae effects showed that Sargassum may affect human thyroid metabolism and thyroid disease [29]. Several brown seaweeds, such as *Laminaria* sp. and *Sargassum* sp. have found containing T3 and T4 organically bound iodine compounds [30]. Brown seaweeds are the only known non-animal sources of thyroid hormones. Eating brown seaweed may provide organically bound iodine as thyroid hormones [30].

### Blood antibody titers

Effects of Sargassum meal supplementation on blood antibody titer of layers are shown in Table 6. There was no difference in blood IgA and IgG among groups. Blood IgM content of 1% and 2% Sargassum meal supplementation groups were higher than that of the other groups (p<0.05). Layers fed 3% and 5% Sargassum meal had a lower IgM content, but the 5% group tended to increase their IgG and IgA titers (p>0.05). High levels of IgG, IgA, and IgM has a positive effect on the defense against bacteria and virus invasion [31]. IgM comprises 5% to 10% of total antibody titer in blood and lymphatic fluid. Under infection, the immune system firstly produces IgM antibody which induce the other immune cells to destroy intruders. Hence, the IgM appears as the first line of defense in the response to an antigen [32]. Brown seaweeds contain polysaccharide of natural sources, which can promote phagocytosis, lymphocytes, and lysosomal enzyme activity [33]. Seaweeds also contain glycosides. Research has shown that glycosides may be related to promotion of immune-stimulating activities, enhancement of immune functions, protection from prolonged heat-induced oxidative stress, antioxidant, and slight immune-stimulating activities [34]. Saraswati’s review [35] indicated that Sargassum was found to modulate the inflammatory response by inhibiting NF-kB and MAPK activation, inhibit inflammation-associated enzymes and scavenge radical species directly as a source of anti-inflammatory agents. In addition, Srivastava and Pandey [36] and Attia et al [37–40] indicated that medicinal plants and their extracts may act as immunostimulant by enhancing non-specific immunity in combating the pathogens. Sargassum hemiphyllum, used in this experiment, may enhance protection from infectious diseases [36].

## CONCLUSION

The diets including 1% to 3% Sargassum meal formulated to isocaloric and isonitrogenous have shown to increase egg production and egg mass of Leghorn layers. However, high supplementation (5%) of Sargassum meal could negatively affect laying performance. Considering the performance of egg production, egg quality, consumers’ acceptability, and immunity stimulation, appropriate supplementation of Sargassum meal is suggested to be 2% in diet for Leghorn layers.

## Figures and Tables

**Table 1:** Diet formula and compositions offered to Hy-Line W-36 laying hens in Sargassum meal trial

Items	Sargassum meal (% in diet)				

	0	1	2	3	5
Ingredients (%)					
Yellow corn, ground	61.87	60.95	60.02	59.09	57.22
Soybean meal	25.68	25.55	25.43	25.30	25.05
Fish meal, CP 65%	2.00	2.00	2.00	2.00	2.00
Sargassum meal	-	1.00	2.00	3.00	5.00
Soybean oil	0.60	0.65	0.71	0.76	0.88
Dicalcium phosphate	0.90	0.90	0.90	0.90	0.90
Limestone, pulverized	8.30	8.30	8.30	8.30	8.30
Salt	0.30	0.30	0.30	0.30	0.30
Choline-Cl (50%)	0.05	0.05	0.05	0.05	0.05
DL-methionine	0.20	0.20	0.20	0.20	0.20
Vitamin-mineral premix1)	0.10	0.10	0.10	0.10	0.10
Total	100.00	100.00	100.00	100.00	100.00
Calculated value					
ME (kcal/kg)	2,854	2,850	2,846	2,842	2,840
Available phosphorus (%)	0.29	0.29	0.29	0.29	0.29
Analyzed value (%)					
Crude protein	17.16	17.21	17.12	17.08	17.20
Calcium	3.66	3.75	3.60	3.82	3.78
Total phosphorus	0.57	0.61	0.62	0.64	0.61

CP, crude protein; ME, metabolizable energy.

1) Supplied per kilogram of diet: vitamin A, 16,000 IU; vitamin D_3_, 2,667 IU; vitamin E, 19.9 IU; vitamin K, 2.7 mg; vitamin B_1_, 1.87 mg; vitamin B_2_, 6.4 mg; vitamin B_6_, 2.7 mg; vitamin B_12_, 16 μg; folic acid, 0.53 mg; calcium pantothenate, 26.7 mg; niacin, 40 mg; Fe (FeSO_4_), 53.3 mg; Cu (CuSO_4_·5H_2_O), 10.7 mg; Mn (MnSO_4_·H_2_O), 93.3 mg; Zn (ZnO), 106.7 mg; I (KI), 0.53 mg; Co (CoSO_4_), 0.27 mg; and Se (Na_2_SeO_3_), 0.27 mg.

**Table 2:** Effects of Sargassum meal supplementation on the feed intake and laying performances of Hy-line W-36 laying hens during 36 to 44 wks of age

Items	Sargassum meal (% in diet)					SEM

	0	1	2	3	5	
Feed intake (g/bird/d)	97.48	96.79	95.86	98.13	91.30	1.24
Laying rate (%)	79.75^bc^	82.90^ab^	85.41^a^	83.68^ab^	77.19^c^	1.28
Egg mass (g/d/hen)	45.13^b^	47.78^a^	46.84^ab^	47.25^a^	43.82^b^	0.98
Feed conversion rate (feed intake/egg mass)	2.10	1.98	2.02	2.06	2.16	0.04
Body weight gain (g/hen)	61.79	34.85	43.43	41.80	31.75	13.28

SEM, standard error of the mean.

a–c Means in the same row with different superscripts differs significantly at 5% level (p<0.05).

**Table 3:** Effects of Sargassum meal supplementation on the egg weight and egg quality of Hy-line W-36 laying hens during 36 to 44 wks of age

Items	Sargassum meal (% in diet)					SEM

	0	1	2	3	5	
Avg. egg weight (g)	59.21	60.55	58.63	58.52	59.08	0.75
Egg shell breaking strength (kg/cm^2^)	2.14	2.12	2.13	2.10	2.06	0.10
Shell thickness (μm)	44.88	36.83	35.38	38.03	38.86	4.28
Albumin height (mm)	3.98	4.09	4.08	3.81	3.90	0.26
Shell weight percentage (shell weight/egg weight, %)	13.25	12.74	13.04	12.97	12.98	0.14
Haught unit	63.33	65.23	66.02	61.72	63.29	3.44
Yolk color						
L* value (lightness)	59.93^b^	59.20^b^	61.85^ab^	63.13^a^	61.98^ab^	1.08
a* value (red color)	4.04	4.23	4.18	4.51	4.13	0.32
b* value (yellow color)	41.42	41.99	44.74	45.74	43.80	1.33

SEM, standard error of the mean.

a,b Means in the same row with different superscripts differs significantly at 5% level (p<0.05).

**Table 4:** Effects of Sargassum meal supplementation in diets on the panel test of egg quality

Score	Sargassum meal (% in diet)					Chi-square test (p-value)

	0	1	2	3	5	
Flavor (%)						
1	0.00	0.00	0.00	0.00	0.00	>0.05
2	4.76	0.00	0.00	4.76	14.29	
3	14.29	28.57	19.05	14.29	33.33	
4	19.05	23.81	14.29	38.10	19.05	
5	47.62	23.81	42.86	23.81	23.81	
6	9.52	23.81	19.05	14.29	9.52	
7	4.76	0.00	4.76	4.76	0.00	
Color (%)						
1	0.00	0.00	0.00	0.00	4.76	<0.05
2	0.00	4.76	4.76	19.05	19.05	
3	23.81	4.76	9.52	19.05	23.81	
4	28.57	38.10	14.29	19.05	14.29	
5	19.05	23.81	28.57	19.05	14.29	
6	28.57	23.81	38.10	19.05	23.81	
7	0.00	4.76	4.76	4.76	0.00	
Tenderness (%)						
1	0.00	4.76	4.76	0.00	0.00	>0.05
2	4.76	0.00	0.00	0.00	19.05	
3	9.52	28.57	23.81	19.05	28.57	
4	38.10	14.29	14.29	38.10	19.05	
5	23.81	23.81	33.33	14.29	23.81	
6	23.81	28.57	19.05	23.81	9.52	
7	0.00	0.00	4.76	4.76	0.00	
Acceptability (%)						
1	0.00	0.00	4.76	0.00	4.76	<0.05
2	0.00	0.00	4.76	9.52	9.52	
3	4.76	19.05	9.52	19.05	28.57	
4	28.57	28.57	19.05	23.81	23.81	
5	42.86	23.81	33.33	28.57	23.81	
6	19.05	28.57	19.05	14.29	9.52	
7	4.76	0.00	9.52	4.76	0.00	
Purchase intention (%)						
1	0.00	0.00	0.00	0.00	0.00	
2	0.00	0.00	0.00	0.00	0.00	
3	0.00	0.00	4.76	9.52	23.81	<0.05
4	9.52	14.29	4.76	14.29	19.05	
5	33.33	33.33	28.57	23.81	28.57	
6	52.38	33.33	38.10	33.33	19.05	
7	4.76	19.05	23.81	19.05	9.52	

Scored on a 1 to 7 point scale (7: very tender, intense, or like; 1: very tough, blank, or dislike).

**Table 5:** Effects of Sargassum meal supplementation in diets on blood characteristics of Leghorn layers

Items	Sargassum meal (% in diet)					SEM

	0	1	2	3	5	
Glucose (mg/dL)	206^b^	217^b^	220^b^	229^b^	258^a^	13
Total protein (g/dL)	4.13	4.28	4.59	4.61	4.62	0.22
Blood urea nitrogen (mg/dL)	2.36^c^	3.47^ab^	3.29^b^	3.63^ab^	4.29^a^	0.31
Total cholesterol (mg/dL)	188	190	223	211	216	28
Triglyceride (mg/dL)	415	444	517	498	535	45
Thyroxine (T_4_) (μg/dL)	1.44^b^	1.48^b^	1.71^ab^	1.58^ab^	1.88^a^	0.11
Triiodothyronine (T_3_) (μg/dL)	45.50^b^	52.83^b^	86.00^a^	87.20^a^	88.33^a^	0.95

SEM, standard error of the mean.

a–c Means in the same row with different superscripts differs significantly at 5% level (p<0.05).

**Table 6:** Effects of Sargassum meal supplementation on blood antibody titers of hens

Items	Sargassum meal (% in diet)					SEM

	0	1	2	3	5	
IgA×10^6^ (mg/L)	11.58	12.60	11.86	11.39	13.47	1.01
IgG×10^3^ (mg/L)	411	424	435	393	476	46
IgM×10^3^ (mg/L)	715^bc^	787^ab^	903^a^	640^c^	667^c^	63

SEM, standard error of the mean; Ig, immunoglobulin.

a–c Means in the same row with different superscripts differs significantly at 5% level (p<0.05).
